# SoftPanel: a website for grouping diseases and related disorders for generation of customized panels

**DOI:** 10.1186/s12859-016-0998-5

**Published:** 2016-04-05

**Authors:** Likun Wang, Cong Zhang, Johnathan Watkins, Yan Jin, Michael McNutt, Yuxin Yin

**Affiliations:** Institute of Systems Biomedicine, Department of Pathology, School of Basic Medical Sciences, Beijing Key Laboratory of Tumor Systems Biology, Peking-Tsinghua Center for Life Sciences, Peking University Health Science Center, Beijing, 100191 China; Institute for Mathematical and Molecular Biomedicine, King’s College London, Guy’s Campus, London, SE1 1UL UK; Department of Research Oncology, King’s College London, Guy’s Campus, Great Maze Pond, London, SE1 9RT UK

**Keywords:** Targeted panel, Gene prioritization, Human disorder phenotype similarity, Web service

## Abstract

**Background:**

Targeted next-generation sequencing is playing an increasingly important role in biological research and clinical diagnosis by allowing researchers to sequence high priority genes at much higher depths and at a fraction of the cost of whole genome or exome sequencing. However, in designing the panel of genes to be sequenced, investigators need to consider the tradeoff between the better sensitivity of a broad panel and the higher specificity of a potentially more relevant panel. Although tools to prioritize candidate disease genes have been developed, the great majority of these require prior knowledge and a set of seed genes as input, which is only possible for diseases with a known genetic etiology.

**Results:**

To meet the demands of both researchers and clinicians, we have developed a user-friendly website called *SoftPanel*. This website is intended to serve users by allowing them to input a single disorder or a disorder group and generate a panel of genes predicted to underlie the disorder of interest. Various methods of retrieval including a keyword search, browsing of an arborized list of International Classification of Diseases, 10th revision (ICD-10) codes or using disorder phenotypic similarities can be combined to define a group of disorders and the genes known to be associated with them. Moreover, SoftPanel enables users to expand or refine a gene list by utilizing several biological data resources. In addition to providing users with the facility to create a “hard” panel that contains an exact gene list for targeted sequencing, SoftPanel also enables generation of a “soft” panel of genes, which may be used to further filter a significantly altered set of genes identified through whole genome or whole exome sequencing. The service and data provided by SoftPanel can be accessed at http://www.isb.pku.edu.cn/SoftPanel/. A tutorial page is included for trying out sample data and interpreting results.

**Conclusion:**

SoftPanel provides a convenient and powerful tool for creating a targeted panel of potential disease genes while supporting different forms of input. SoftPanel may be utilized in both genomics research and personalized medicine.

**Electronic supplementary material:**

The online version of this article (doi:10.1186/s12859-016-0998-5) contains supplementary material, which is available to authorized users.

## Background

Deployment of next-generation sequencing is becoming increasingly widespread in scientific research and clinical settings. While whole genome and whole exome sequencing have greatly aided our understanding of the molecular basis of disease, targeted sequencing of a panel of genes offers a number of benefits over these approaches [1, 2]. Chief among these is the issue of unsolicited findings, which are results that are not directly relevant to the initial reason for performing a diagnostic test. Such scenarios may present a host of ethical issues, in particular in whole genome and whole exome sequencing. A second advantage of the targeted sequencing approach is that narrowing the region to be sequenced from the whole genome to a select panel of genes can result in marked savings of cost and time, while obtaining deeper sequencing coverage with a larger number of samples. Giving priority to genes that are potential causes of disease can thus help researchers design a targeted panel that enables investigation of biological or clinical issues both effectively and economically.

A number of approaches have been proposed to prioritize candidate disease-associated genes [3–7]. Among these approaches, one of the most widely-used concepts is the “guilt-by association” principle, which suggests that genes with the same interaction partners or same expression data most likely share the same biological function [8]. According to this general principle, genes associated with the same or similar disorders tend to have a higher probability of having similar functions, as well as similar gene expressions and physical interactions among gene products [9]. Therefore, starting from a set of seed genes known to be related to a particular disorder, candidate genes can be ranked according to their functional similarity to these seed genes. A variety of biological data sources are used to calculate the functional similarities from existing studies, including protein-protein interaction, functional pathways, gene ontology and many others [10]. Based on the fact that these different data sources are complementary in nature, the integration of multiple data sources can improve the coverage and accuracy of prioritization [11]. Most of these approaches call for prior genetic knowledge of the disease of interest in order for a set of seed genes to be submitted as input. However, for a significant proportion of human disorders, the genetic basis remains unknown [12]. Moreover, for clinicians with a limited knowledge of genetics and molecular biology, the selection of an appropriate set of seed genes can be a particularly daunting task.

Given the absence of a known genetic basis to many human diseases, some gene prioritization tools have been devised using the similarity of a disease’s phenotypic profile to that of other diseases or text mining methodology [5, 13, 14]. Many human disorders are modular in nature [15]. This means that the genetic mechanisms and phenotypes observed in one disease may also be operative in other diseases with a similar phenotype. Lage et al. investigated this modularity at the phenotypic level by text mining and integrated the resultant phenotypic similarity profiles with a protein-protein interaction (PPI) network [16]. Wu and colleagues used the correlation between phenotype similarity profiles and gene proximities in a PPI network to quantify the association between a disease and a gene [5]. The incorporation of phenotypic similarity profiling improved the performance of these gene prioritization methods. The most widely used dataset profiling phenotypic similarity was published by Driel et al.[17]. However, this dataset covers only 5080 diseases, which is substantially fewer than the 7000 diseases listed in the Online Mendelian Inheritance in Man (OMIM) database, thus limiting the coverage and accuracy of the methods that make use of the Driel et al. dataset [12].

In an attempt to overcome these limitations, we have developed a clinically oriented candidate disease gene prioritization tool called *SoftPanel*. Multiple retrieval methods are supported and can be combined to help users get a disorder group and a set of candidate genes, with little or no prior genetic knowledge required. Due to its familiarity to clinicians and the fact it reflects connections between similar disorders and their underlying genes, we used indices from the International Classification of Diseases, 10th revision (ICD-10) to help identify disorders of interest. Searching by keyword as well as other advanced options were implemented by making use of OMIM application programming interface (API) functions. In addition, we measured the phenotypic similarity between human disorders with the newly updated OMIM database and attempted to improve its performance by comparing different approaches to weighting, as well as by adding the parsing of the title portion rather than just the textual description [17]. Various types of data resources were integrated in our prioritization method, including PPI networks, pathways from the Kyoto Encyclopedia of Genes and Genomes (KEGG), the REACTOME and Gene Ontology (GO) databases, as well as other functional gene annotations [18–20]. An online support vector machine (SVM) was applied to identify genes that potentially underlie a given disease group by employing gene set enrichment analysis (GSEA) of known disease-associated genes. The output provided includes a list of potential disease-associated genes ranked by significance.

## Implementation

### Retrieval strategies

The website provides an arborized ICD-10 index list for selecting and grouping disorders. The corresponding relationship between ICD-10 index codes and OMIM record numbers was derived mainly from the OMIM and Orphanet databases [21]. The data were manually checked and added. A network of disorder similarities was used to find disorders similar to the disorder of interest. Further details regarding the construction of the disorder phenotype similarity network are provided below. Searching for disorders with specific keywords invokes the APIs provided by the OMIM database resulting in an automatically search and extraction keyword-matching disorders. Some advanced search options are presented on this website. First-time users of this function can register for an API key for free on the OMIM database.

### Construction of the phenotypic similarity matrix

The OMIM database contains more than 20,000 records, each of which describes a gene or disorder. OMIM records are prefixed with one symbol out of a possible five, with each symbol corresponding to a particular type of record. Only records prefixed with “#”, “%” and “none” representing phenotypes of possible disease conditions (rather than those linked to certain genes) were selected. The text (TX) and clinical synopsis (CS) fields were then used to construct textual sub-vectors, and the title field (TI) was used to construct the title sub-vector. MetaMap was used to map the concepts contained in biomedical text to entries in the Unified Medical Language System (UMLS) Metathesaurus (MTH) and Medical Subject Headings (MeSH) [22]. Semantic types were thus restricted to “anatomy”, “chemicals and drugs”, “disorders”, “genes and molecular sequences”, and “physiology”. Before parsing the title field, serial numbers or letters referring to a subtype were discarded to maintain the main characteristic of the disorders. Next, the textual sub-vector and title sub-vector corresponding to one given record were added together. Records that could not be mapped to any concept in both the title and textual sections were eliminated from further analysis. Concepts occurring in one record only were also discarded in order to improve the performance of the similarity network and reduce calculation time. To account for the generic applicability of certain concepts, terms that appeared across a range of diverse phenotypes but did not provide further information about the phenotype described were manually removed; for example, “disease”.

OMIM records with relatively long textual description tend to contain more concepts. In an effort to correct the differences among vectors caused by different OMIM record lengths, we applied the following global weighting to each concept:

$$ \mathrm{Global}\ \mathrm{weighting}={ \log}_2\left(\frac{N}{n_{concept}}\right) $$

where *N* is the total number of records, and *n*_*concept*_ is the total number of records containing a specific concept. We also defined local weighting as follows:

$$ \mathrm{Local}\ \mathrm{weighting}=0.5 + \frac{r_{concept}}{2\times {r}_{max}} $$

where *r*_*concept*_ is the frequency of a specific concept in a record, and *r*_*max*_ is the frequency of the concepts appearing most frequently in a record. Similarity was calculated by finding the cosine of the angle between each pair of record vectors. The comparative analysis of the different approaches to weighting is discussed below in the Results section.

### Preparation of benchmark datasets

To evaluate the performance of our similarity matrix, two sets of OMIM record pairs that were known to overlap with respect to phenotype were sought as our benchmark datasets.

First, we employed the “Phenotypic Series” dataset, which consists of a tabular view of the genetic heterogeneity of similar phenotypes across the genome compiled from the OMIM database. Each series collects all the subtypes of a particular disorder, such that the subtypes are phenotypically similar to each other with only a few genetic or clinical distinctions. Two subtypes from the same phenotypic series were considered phenotypically similar. Pairing of subtypes in this way resulted in retrieval of a total of 26,618 disorder pairs.

To improve the robustness of our results, an alternative, previously described benchmark dataset referred to as the “Linked OMIM Record Pairs” dataset, was also used [16]. It leverages on those records that embed a reference to another record. For example, if the description of record A references record B, this is quite probably a result of the high phenotypic overlap between the two. Such links between OMIM records were extracted and restricted to those prefixed with “#” or “%” to yield 16,155 pairs of disorder records. To determine the prevalence of false-positive pairs in this link-derived dataset, 100 random pairs were extracted and manually checked for phenotypic similarity. Of these 100 random pairs, 82 pairs turned out to be true positives, that is, were disorder pairs with actual phenotypic overlap.

### Performance evaluation of phenotype similarity matrices

Receiver operating characteristic (ROC) curves were used to evaluate the performance of phenotype similarity matrices using the ROCR package in R [23]. If a record pair in the similarity matrix was present in a given benchmark dataset, we labeled that pairing as a true positive, and the remainder in the matrix were considered false positives.

### Gene set enrichment analysis

After identifying disorders with a similar phenotypic profile, genes known to be associated with this grouping of disorders are automatically extracted from the OMIM database and used as input to GSEA. Two supporting sub-databases are available to SoftPanel users for performing online GSEA. The first sub-database combines PPI data from the PINA, iRefIndex and STRING databases [24–26]. All genes that can interact with a given gene are treated as a gene set. Based on this gene set and the original list of disease-associated genes, a *p-value* is calculated with a one-sided Fisher's exact test. This *p-value* is used to evaluate the likelihood of PPIs among the gene of interest and the original disease-associated genes. This test is performed on all other potentially associated genes that were not included in the original gene list. The second sub-database contains the gene set collections from the MSigDB database [27, 28]. MSigDB gene sets are divided into several major collections, each of which contains gene sets generated from different resources. GSEA is performed on all gene sets that belong to user-selected collections. In this website, gene sets are displayed and ordered by respective *p-values* for each user-selected collection.

### Prediction of underlying genes

After GSEA, a *p-value* can be assigned to a gene in order to estimate the degree of PPI between this gene and the original disease-associated genes. In addition, a given gene may belong to several gene sets in a certain MSigDB collection. Gene set enrichment *p-values* for those gene sets that refer to the original disease-associated gene list can be calculated as described above. For a given MSigDB collection (such as all gene sets from the KEGG database), the lowest GSEA *p-value* among these gene sets (to which the given gene belongs) is also assigned to this given gene. Under normal circumstances, GSEA is performed on several major collections. As such, several collection-based scores may be assigned to a gene. All of these scores are then used as the input for performing machine learning. The original disease-associated genes are treated as the positive training set, and a certain number of genes that are not in the original disease-associated gene list are randomly selected as the negative training set. In practice, some genes in the original disease-associated gene list do not have significant interactions with other genes in this list, and do not belong to significant gene sets. These genes cannot provide useful information for machine learning, and are therefore removed from the core gene list. To ensure that the machine learning process is real-time and carried out online, this website employs Support Vector Machine in JavaScript (svmjs), which supports arbitrary kernels.

## Results

### SoftPanel workflow

Researchers and clinicians frequently wish to investigate a series of disorders, rather than a single disease. These disorders may share similar phenotypes and thus be difficult to distinguish. Hence, in clinical practice, one effective strategy is to package disorders with similar phenotypes into a targeted sequencing panel. To facilitate this process, we offer the SoftPanel website, which is used to group and classify disorders with similar phenotypes in order to identify known and predicted disease-associated genes.

**Fig. 1:**
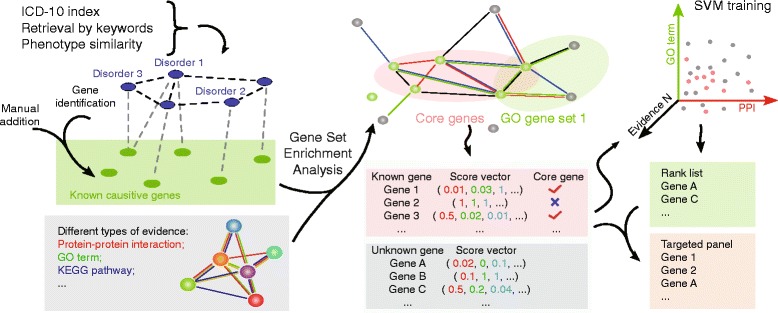
Flow chart for this website. Users first define a group of disorders using phenotype keyword searches, disorder phenotype similarity scoring, ICD-10 index specification, or manual editing. After determining a disorder group, known disease-associated genes are extracted from the OMIM database automatically. Users can next perform gene set enrichment analysis (GSEA) on the extracted list of disease-associated genes. Finally, an online supporting vector machine (SVM) is applied to refine the identification of genes that potentially underlie the disorder of interest

This website provides several options for grouping disorders (Fig. 1). First, SoftPanel gives users the opportunity to group disorders using ICD-10 indices. An arborized ICD-10 index list is displayed on the website so that users can select and group disorders by choosing the corresponding ICD-10 indices. After one or more ICD-10 indices are selected, all OMIM numbers for disorders of this class are extracted. The second option makes use of a disorder similarity matrix of 7995 diseases, which we constructed by employing text mining methods on records from the OMIM database. Upon inputting the OMIM number of a particular disorder, a list of similar disorders is returned, and this list is ranked by similarity score. In addition, the overlapping phenotype keywords for these disorders are also displayed for reference. Third, users can search for disorders by inputting specific keywords, which in turn invokes the APIs provided by the OMIM database. Users can then select disorders of interest and add them to the working group. All the above methods of retrieval can be combined to either define a broader group of disorders or refine disorders into more closely related subgroups. Once a group of disorders is generated, the genes associated with these disorders are extracted and displayed in a table. Users can manually add or delete genes from this table as desired to form the original gene list.

Online GSEA is then performed based on two supporting sub-databases, and use of the original gene list as input. One sub-database integrates PPI data from the PINA, iRefIndex and STRING databases, and covers 19,903 genes. The other sub-database contains seven major gene collections from MSigDB. A *p-value* is thus assigned to a gene in order to estimate the degree of PPI or one of the seven types of functional similarities between this gene of interest and the original disease-associated genes. All of these scores are then used to construct an eight-dimensional vector, which is used as the input for performing machine learning. Support vector machines were employed to produce the ranked list of potential genes.

### Construction and evaluation of the disorder phenotype similarity matrix

We measured the similarities among 7995 human phenotypes recorded in the OMIM database. Metamap was employed to parse the disorder record and link concepts contained in the title and textual description to entries in UMLS MTH and MeSH. The title of a record may reveal the main characteristic of a disorder. In order to group disorders with similar characteristics, the serial numbers of subtypes for a particular disorder listed in the title field were discarded before parsing. Addition of the title portion of the records improved the performance of our similarity matrix (see Additional file 1: Figure S1). Next, the feature vectors were weighted and phenotypic similarities between OMIM record pairs were quantified by calculating the cosines of the angles between each pair of record vectors.

**Fig. 2:**
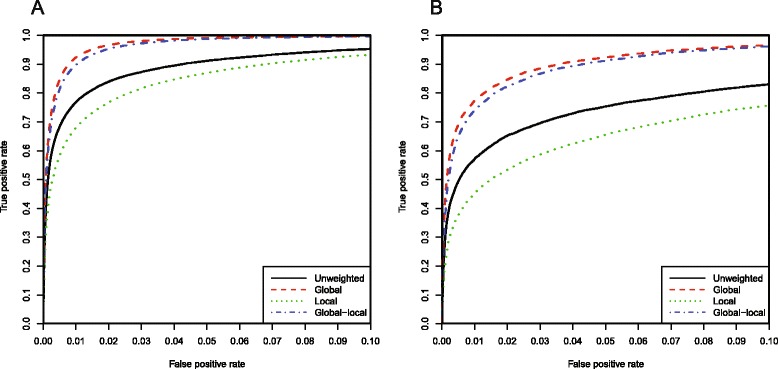
ROC curves of phenotype similarity matrices with different methods of weighting. ROC analysis with the two benchmark datasets (**a**: Phenotypic Series, **b**: Linked OMIM Record Pairs) showed that global weighting was superior to other forms of weighting. The range of false positive rates was restricted to (0, 0.1) in order to highlight the differences between each curve more clearly

In order to improve the performance of our method, we explored different approaches to weighting and chose the one with the best performance. Four sets of similarity score matrices with different weightings (unweighted, global weighting, local weighting, and global–local weighting), were compared by ROC curves against the two benchmark datasets (Phenotypic Series and Linked OMIM Record Pair datasets). This ROC analysis revealed that globally-weighted vectors outperformed other weighting methods, showing the greatest concordance with our reference datasets, and producing a significantly higher area under the curve (AUC) (Fig. 2, Table 1 and Additional file 2). As such, only global weighting was employed in our SoftPanel similarity matrix.

**Table 1:** AUCs using matrices with different weightings and two benchmark datasets

	Unweighted	Global	Local	Global–local
Phenotypic Series	0.983	0.996	0.976	0.995
Linked OMIM Record Pairs	0.945	0.985	0.923	0.982

Four sets of similarity score matrices with different weightings (unweighted, global weighting, local weighting, and global–local weighting) are shown. The method applying global weighting is used in SoftPanel

We computed a ROC curve for our SoftPanel similarity matrix (Table 1). The Phenotypic Series-defined pairs and Linked OMIM Record Pairs were separately classified as true positives, while the rest were deemed false positives. In both conditions, the AUC was high (0.996 against the Phenotypic Series and 0.985 against the Linked OMIM Record Pairs dataset), demonstrating that our similarity matrix is able to distinguish between similar and dissimilar pairs of disorders. The majority of the false positives possessed relatively low similarity scores (most < 0.05). However, the actual false-negative and false-positive rates may be even lower since not all record pairs in the benchmark datasets are phenotypically overlapped, and some positive pairs resemble each other to only a limited extent, while there are some false positives which actually have similar phenotypes but for various reasons may not be recorded in the benchmark datasets.

To test further the reliability of our similarity matrix, all phenotypically overlapping record pairs in our similarity matrix were divided into 10 intervals according to their similarity scores. For each interval, we calculated the fraction of record pairs that overlap with the true positive dataset (see Additional file 3: Figure S2). The positive correlation when evaluated against both benchmark datasets suggested that the higher the phenotypic similarity score between two specific records measured in our matrix, the higher the probability that these two records were considered to have phenotypic overlap by the OMIM curators. This analysis provided evidence for the utility and reliability of the similarity score as a measure of phenotypic overlap between two disorder records.

### Comparison with previous methods

**Fig. 3:**
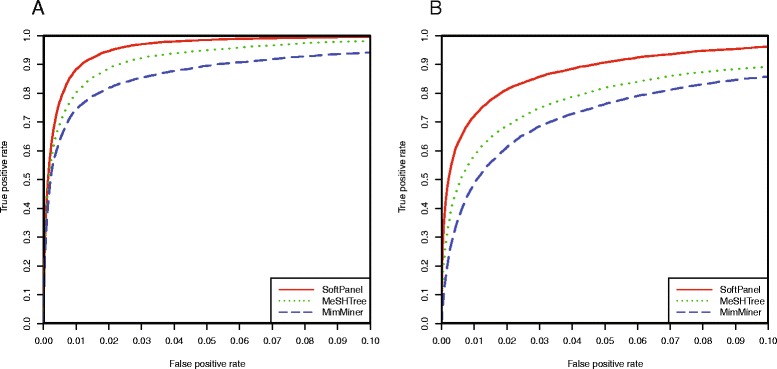
ROC curves of SoftPanel and MimMiner phenotype similarity matrices. ROC analysis with the two benchmark datasets (**a**: Phenotypic Series, **b**: Linked OMIM Record Pairs), demonstrated that our similarity matrix (SoftPanel) performed better compared with the results of an existing method (MimMiner) and a similarity matrix constructed as MimMiner using the newest OMIM database (MeSHTree). The range of false positive rates was restricted to (0, 0.1) in order to highlight the differences between each curve more clearly

To assess whether our approach was superior to that of existing prioritization methods that integrate disease phenotype similarity data, we downloaded the MimMiner network matrix dataset [17] and compared it with a SoftPanel similarity matrix using ROC curves against the two benchmark datasets. In the first instance prior to calibration, the matrices were restricted to those with identical dimensions in order to enable a consistent comparison. We found the SoftPanel similarity matrix performed better than results from MimMiner (Fig. 3, Table 2 and Additional file 2). To investigate the influence of an updated OMIM database on MimMiner, we employed the tree structure of MeSH to parse records from the newest OMIM database, using the concepts from the anatomy and disease categories, and weighted the vectors with the global–local weighting method as described in MimMiner. Again, our approach outperformed the updated similarity matrix (designated as MeSHTree) which was constructed with the newest version of OMIM database just as the approach adopted by MimMiner (Fig. 3, Table 2 and Additional file 2). In addition to using the newest OMIM database to obtain greater coverage of human disorders, we also improved the performance of the phenotype similarity matrix by improving its means of construction.

**Table 2:** AUCs using different similarity matrices and two benchmark datasets

	SoftPanel	MeSHTree	MimMiner
Phenotypic Series	0.995	0.988	0.972
Linked OMIM Record Pairs	0.981	0.961	0.944

SoftPanel: our similarity matrix. MeSHTree: a similarity matrix constructed as MimMiner using the newest OMIM database. MimMiner: data was downloaded from the MimMiner website. Prior to calibration, the matrices were restricted to those with identical dimensions in order to enable a consistent comparison. Hence, the results of SoftPanel in Tables 1 and 2 are different

### Case study: prediction of genes underlying epilepsy-related disorders

Once a group of disorders is generated, the genes known to be associated with these disorders are extracted and displayed in a table. Users can then predict potentially novel disease-associated genes using the original disease-associated genes.

**Fig. 4:**
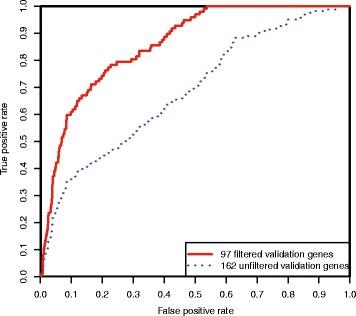
ROC curves for validation in a case study using epilepsy as the disorder of interest. Two ROC curves were drawn according to the predicted rankings of the genes in the unfiltered and filtered validation datasets

To evaluate the performance of this prediction method, we searched the OMIM database for the keyword “epilepsy”. This returned a total of 494 OMIM phenotype description entries and 354 OMIM gene description entries. Despite the fact that some of these OMIM entries had no obvious relationship with epilepsy, we retained all of these hits for evaluation. A set comprising 391 known epilepsy disorder-related genes was then compiled by extracting the genes linked to the 494 OMIM phenotype description entries. This original disease-associated gene set was used as the positive training dataset. From the original 354 OMIM gene description entries, we extracted 345 unique genes, 183 of which had already been extracted from the phenotype description entries. We therefore used the remaining 162 genes as our initial, unfiltered positive validation dataset. The proportion of false positive genes in this unfiltered positive validation dataset may be high due to our retention of all OMIM search hits and their absence from the phenotype description entries. Therefore, we investigated and found that certain genes in this validation dataset had higher PPI likelihoods with genes outside the original disease-associated gene set, than with genes within the original gene set. These genes therefore had a high probability of being false positives, and so we removed them to give a filtered validation dataset of 97 genes. Two ROC curves were drawn according to the predicted ranking of the genes in the unfiltered and filtered validation datasets (Fig. 4).

## Discussion

We have provided an online real-time web server called SoftPanel that groups disorders and predicts genes potentially underlying these disorders. One of the most obvious applications of this tool would be the generation of customized panels of genes that can be used as input for a targeted next-generation sequencing investigation. This website can group or classify disorders, extract disease-associated genes, and predict which additional genes may be associated with the disorder of interest in a semi-automated manner. All the methods used in this website have been evaluated and rigorously tested for their accuracy and reliability in generating panels of *bona fide* disease-associated genes.

One of the advantages of our method is that it takes the needs of the user into consideration. Compared to existing gene prioritization methods, we allow the user to begin with a disorder of interest rather than with a pre-determined set of seed genes. This is based upon our observation that the former scenario is the most frequent starting point for clinicians and researchers interested in a given condition.

Human disorder phenotype similarity profiles have been used in previous studies [5, 29, 30]. We have supplemented this approach with the addition of the title portions of OMIM records, as they can reveal the main characteristics of diseases. At the same time, we applied global weighting to the vectors instead of the global–local weighting used in previous work [16, 17]. ROC curves demonstrated the superiority of the similarity matrices generated by SoftPanel over those generated by existing methods.

There have been several open-source tools designed to collect and analyze phenotypic information for patients with genetic disorders. One of these is PhenoTips, which is a well-developed method and has an intuitive user interface [7]. PhenoTips allows for the collection, classification, and analysis of phenotypic information and uses the Human Phenotype Ontology. One of the intentions of grouping disorders using phenotypic information in SoftPanel is to output seed gene lists for the purpose of gene prioritization, something that complements rather than replicates the capabilities of PhenoTips. PhenoTips may be used to help clinicians make accurate diagnoses but it does not delve deep enough into the genetic or molecular basis of disorders. SoftPanel, however, is able to provide a list of both known and unknown potential disease-causing genes that can then be used by clinicians to customize a panel for gene screening or identifying rare driver genes. Thus, SoftPanel differs from tools like PhenoTips in its purpose, design, and user orientation.

More importantly, SoftPanel offers great convenience for researchers who wish to evaluate genes that have been identified from microarray or RNA-sequencing data as being differentially expressed in their phenotype or condition of interest as compared with healthy tissue or some other reference condition. Not infrequently, the list of differentially expressed genes is quite long, making it difficult to experimentally validate the correlations between key genes and human disease phenotypes. SoftPanel, however, allows users to identify a set of core genes that contribute to a condition by using gene set enrichment analysis and the supporting machine vector approaches. The output genes are ranked according to their significance, and users can then compare them with a list of genes ordered by significance of differential expression or any other metric of interest. SoftPanel thus enables *in silico* validation, filtering and prioritization of gene lists that have been acquired through alternative approaches such as differential gene expression analysis.

In summary, we propose that combining PhenoTips’ specific capabilities with SoftPanel’s capabilities would greatly broaden the scope of investigation, improve user experience, and produce specific information relevant to examining the relationship between genotypes and disease phenotypes in humans.

## Conclusions

SoftPanel is capable of generating convenient and high-utility customized gene panels, oriented towards clinical practice and *in silico* validation of candidate genes for researchers. The availability of multiple retrieval methods gives researchers and clinicians great flexibility and eliminates the requirement for prior genetic knowledge. With its breadth of function and ease of use, this website will be a useful resource to both the clinical and biomedical research communities.

## Availability and requirements

Project name: SoftPanel

Project home page: http://www.isb.pku.edu.cn/SoftPanel/

Operating system(s): Platform independent

Programming language(s): HTML, JavaScript, R

Other requirements: none

License: GNU GPL

Any restrictions to use by non-academics: no.

## Additional files

Additional file 1: Figure S1. ROC curves of phenotype similarity matrices constructed with or without title portions. ROC analysis with the two benchmark datasets (A: Phenotypic Series, B: Linked OMIM Record Pairs) suggested that the similarity matrix constructed with both the text and title portions of OMIM records outperformed the matrix constructed with the text portion only. The range of false positive rates was restricted to (0, 0.1) in order to highlight the differences between each curve. (PDF 270 kb) Additional file 2: In additional to the ROC curve noted in the “Implementation” section, we also calculated ROC curves in an alternative way in order to compare the differences between different similarity matrices. (PDF 73 kb) Additional file 3: Figure S2. Fraction of record pairs that overlap with the two benchmark datasets. All phenotypically overlapping record pairs in our similarity matrix were divided into 10 intervals according to their similarity scores. For each interval, we calculated the fraction of record pairs that overlapped with a given benchmark dataset (A: Phenotypic Series, B: Linked OMIM Record Pairs). This analysis indicated that the similarity score is a useful and reliable measure of phenotypic overlap between two disorder records. (PDF 260 kb)

## Abbreviations

API: application programming interface
GO: gene ontology
GSEA: gene set enrichment analysis
ICD-10: international classification of diseases, 10th revision
KEGG: Kyoto encyclopedia of genes and genomes
OMIM: online Mendelian inheritance in man
MeSH: medical subject headings
MTH: metathesaurus
PPI: protein-protein interaction
ROC: receiver operating characteristic
SVM: support vector machine
UMLS: unified medical language system
