# Supplementary figures and images for: SoftPanel: a website for grouping diseases and related disorders for generation of customized panels
Source: BMC Bioinformatics. 2016 Apr 5;17:153. doi: 10.1186/s12859-016-0998-5 (PMC4820874; doi:10.1186/s12859-016-0998-5)

A

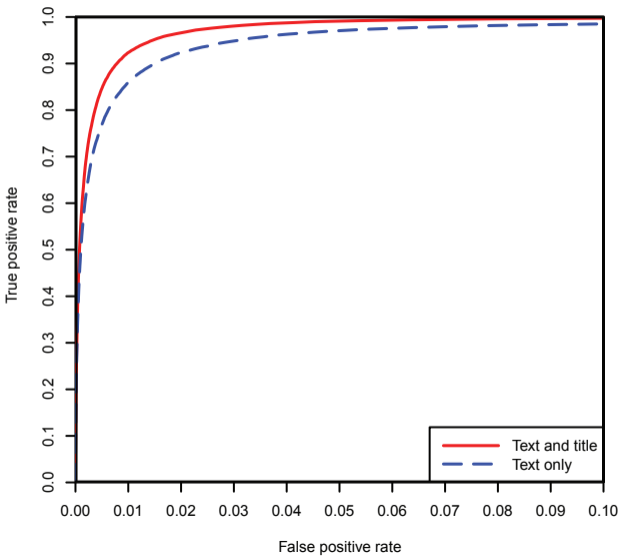

B

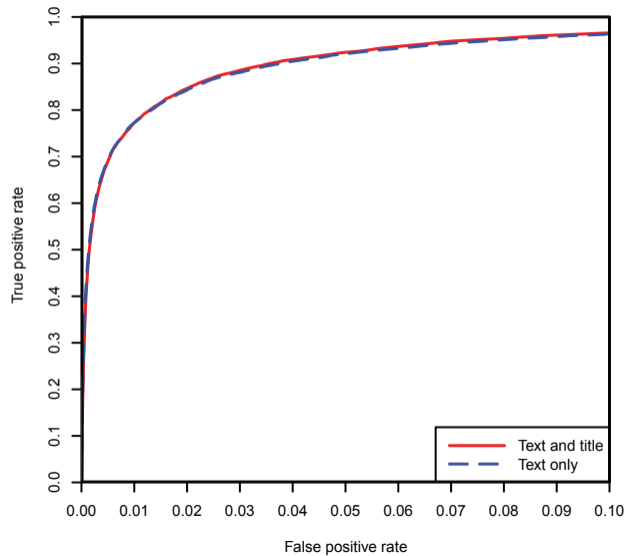

Supplement: Additional file 1: Figure S1. — ROC curves of phenotype similarity matrices constructed with or without title portions. ROC analysis with the two benchmark datasets (A: Phenotypic Series, B: Linked OMIM Record Pairs) suggested that the similarity matrix constructed with both the text and title portions of OMIM records outperformed the matrix constructed with the text portion only. The range of false positive rates was restricted to (0, 0.1) in order to highlight the differences between each curve. (PDF 270 kb) [file 12859_2016_998_MOESM1_ESM.pdf]

**A**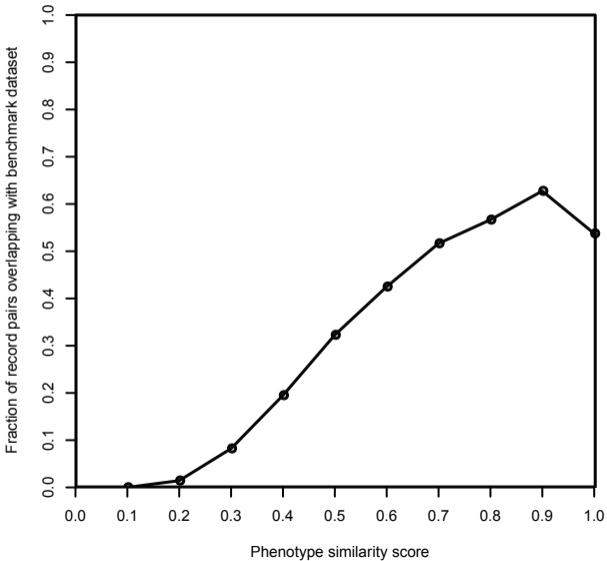**B**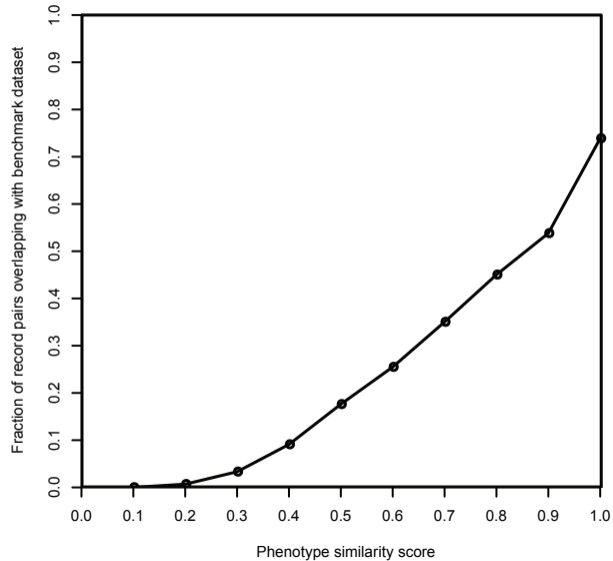

Supplement: Additional file 3: Figure S2. — Fraction of record pairs that overlap with the two benchmark datasets. All phenotypically overlapping record pairs in our similarity matrix were divided into 10 intervals according to their similarity scores. For each interval, we calculated the fraction of record pairs that overlapped with a given benchmark dataset (A: Phenotypic Series, B: Linked OMIM Record Pairs). This analysis indicated that the similarity score is a useful and reliable measure of phenotypic overlap between two disorder records. (PDF 260 kb) [file 12859_2016_998_MOESM3_ESM.pdf]
