# Supplementary material for: SoftPanel: a website for grouping diseases and related disorders for generation of customized panels
Source: BMC Bioinformatics. 2016 Apr 5;17:153. doi: 10.1186/s12859-016-0998-5 (PMC4820874; doi:10.1186/s12859-016-0998-5)
Supplement: Additional file 2: — In additional to the ROC curve noted in the “Implementation” section, we also calculated ROC curves in an alternative way in order to compare the differences between different similarity matrices. (PDF 73 kb) [file 12859_2016_998_MOESM2_ESM.pdf]

Single disease ROC curve

In additional to the ROC curve noted in the "Implementation" section, we also calculated the ROC curves in an alternative way to compare the differences between similarity matrices. The ROC curve described in the "Implementation" section puts all resembled record pairs in one positive set, and treats all pairs as a whole set. Each similarity matrix should have two ROC curves corresponding to the two benchmark datasets. We also use another method to compare the differences between similarity matrices, which is referred to as the "single disease ROC curve". This method first selects one record from a Phenotypic Series, and treats all the remaining phenotype records (7994 in total) as the whole set. Only the records from the same Phenotypic Series as the selected one were treated as true positives in one calculation. Thus ROC curve testing for each similarity matrix was performed 2453 times, equaling the number of records in all the Phenotypic Series. When comparing SoftPanel with MeSHTree and MimMiner, selected records were restricted to those appearing in the MimMiner matrix. As such, ROC curve testing for each matrix was performed 904 times. When comparing the performance of two similarity matrices, we performed a paired Student's *t*-test on the AUCs for the two matrices to verify the significance of any difference.

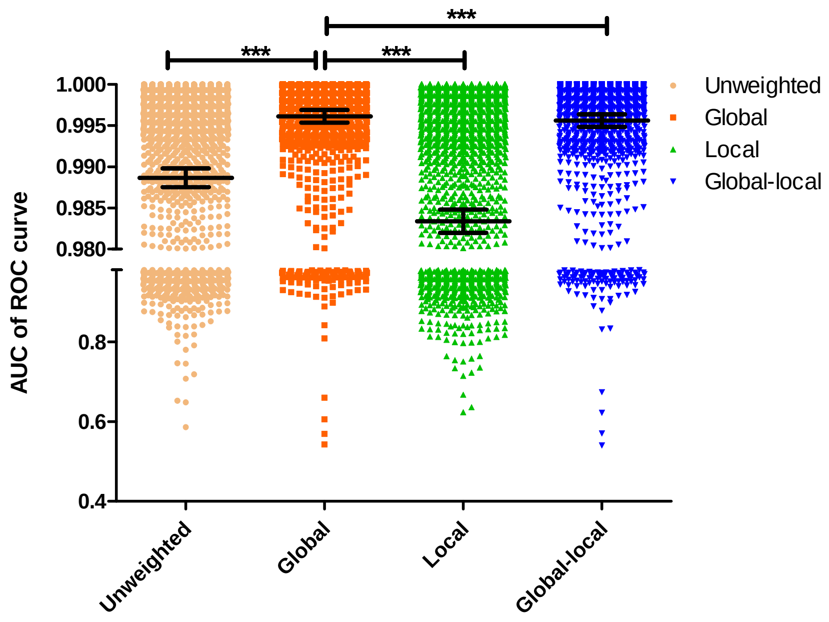

Figure S3 AUCs from single disease ROC curves using matrices with different weightings. AUCs from single disease ROC curves are plotted as scatter plots and the mean and 95% confidence intervals are indicated by lines. Differences between each of two matrices were analyzed with the Student's *t*-test. Statistical analysis demonstrated that the global weighting approach is significantly superior to other forms of weighting (p-value: Global versus Unweighted: 1.14×10^{-69}; Global versus Local: 4.82×10^{-97}; Global versus Global-local: 1.42×10^{-40}).

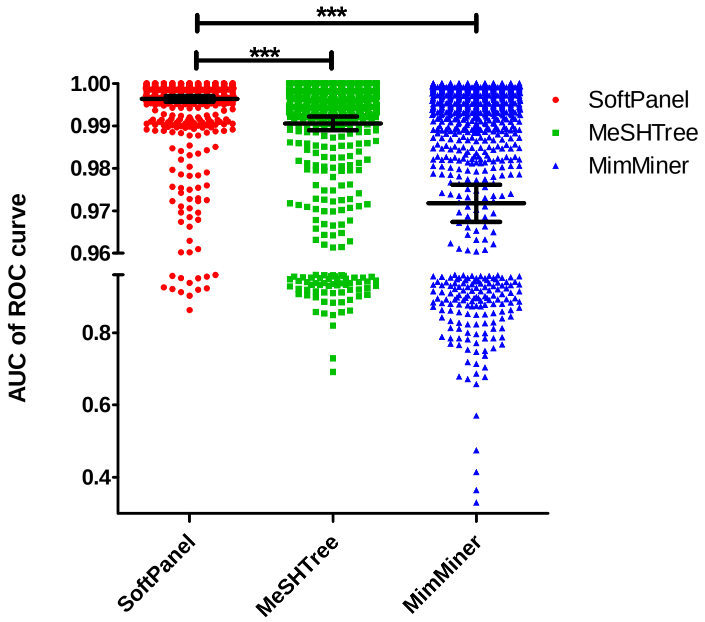

Figure S4 AUCs from single disease ROC curves using different similarity matrices. Single disease ROC curves showed that our similarity matrix (SoftPanel) outperformed MimMiner and MeSHTree. AUCs are presented with means and 95% confidence intervals, and were analyzed with the Student's *t*-test (p-value: SoftPanel versus MimMiner: 1.39×10^{-28}; SoftPanel versus MeSHTree: 8.34×10^{-18}).
